# Conjunctival Lesions: A 5-Year Basic Demographic Data and Clinicopathological Review in a Tertiary Eye Care Hospital

**DOI:** 10.1007/s44197-021-00017-1

**Published:** 2021-11-30

**Authors:** Hind M. Alkatan, Khalid M. Alshomar, Hala A. Helmi, Wajda M. Alhothali, Abdulaziz M. Alshalan

**Affiliations:** 1 Department of Ophthalmology, College of Medicine, King Saud University, Riyadh, Saudi Arabia; 2 Department of Pathology and Laboratory Medicine, College of Medicine, King Saud University, Riyadh, Saudi Arabia; 3 King Saud University Medical City, King Saud University, Riyadh, Saudi Arabia; 4 Ad Diriyah Hospital, Riyadh Third Health Cluster (Ministry of Health), Ad Diriyah, Saudi Arabia

**Keywords:** Conjunctiva, Ocular surface squamous neoplasia (OSSN), Melanocytic, Epidemiology, Caruncle

## Abstract

**Background:**

Conjunctival lesions are common with a wide spectrum of benign, premalignant, and malignant lesions. Few histopathological studies have been conducted on conjunctival lesions with variable designs and results. Our aim in this study is to provide information on common conjunctival lesions seen in an ophthalmology tertiary care center in Saudi Arabia.

**Methods:**

A retrospective, observational study of all consecutive conjunctival tissue specimens sent for histopathological assessment to the pathology department from 2015 to 2019 were analyzed. Clinical data were collected from medical records, and the histopathological slides were reviewed by a single pathologist.

**Results:**

A total of 110 conjunctival specimens from 108 patients were included (mean age: 53 years, 67 males and 43 females). Bilateral involvement was mostly found in inflammatory lesions (40%). Most lesions were benign (91%), with a significantly longer duration of symptoms in malignant lesions (*p* = 0.036*). The clinical diagnosis matched the final histopathological diagnosis in 75.5% of the total specimens. The most frequent category of benign lesions was fibrodegenerative and proliferative lesions (53.6%), with a significantly higher prevalence among adult males (*p* < *0.001*). Melanocytic lesions were more common in children (33.3%) than adults (9.8%), and the mean age of children was significantly lower (*p* = *0.013*). The most frequent malignant lesion was ocular surface squamous neoplasia (50%), with equal prevalence among males and females. The overall outcome was favorable in 89.4% and unfavorable in 10.6%, mostly due to surgical complications, further progression of the lesion, or recurrence.

**Conclusion:**

This study shows variability in the frequency of conjunctival lesions based on gender, age, geographical, racial, and environmental factors. There has been a shift in the gender-based prevalence of ocular squamous neoplasia over the last three decades, probably due to a change in lifestyle.

## Introduction

The conjunctiva is a thin, transparent membrane that covers the inner surface of the eyelids and anterior sclera and provides a protective barrier for the eyes. A broad spectrum of lesions may arise from the conjunctiva, including the caruncle, ranging from benign lesions, such as pterygium, solid dermoid, nevus, and pyogenic granuloma, to premalignant ocular surface squamous neoplasia (OSSN) and aggressive malignancies, such as malignant melanoma and squamous cell carcinoma (SCC) [1–5]. The type and prevalence of conjunctival lesions vary depending on age, race, immunity, and chronic sun exposure [2, 4–6].

There is a relative paucity of large, published studies documenting conjunctival lesions histopathologically. In a large, published study of conjunctival tumors in an ocular oncology tertiary care referral center, conjunctival tumors were found to be benign (52%), premalignant (18%), or malignant (30%) [7]. Of the 18 broad tumor types reported, the three most common lesions included in all age groups were nevus (23%), OSSN (14%), and primary acquired melanosis (PAM) in 12% [7]. A recent study describing conjunctival biopsy specimens from Turkey demonstrated that nonneoplastic lesions, namely, pterygium, were the most frequently encountered lesion (54.78%) [2]. In the neoplastic group, benign lesions, such as nevi, were the most common (9.5%), whereas the most common malignant neoplastic lesion was SCC (3.5%) [2]. Another similar study in the Egyptian population reported that pyogenic granulomas (30.7%) and nevi (22.9%) (mostly compound nevi) were the most frequent histologically diagnosed conjunctival lesions with correct clinical diagnosis in most cases [1].

Recognition of conjunctival tumors, tissue diagnosis, and understanding of predisposing factors are crucial. This study aimed to compare the information about common conjunctival lesions in a large ophthalmology tertiary care center in Saudi Arabia to other international reports using a histopathological database.

## Materials and Methods

We retrospectively reviewed a cohort of all conjunctival tissue specimens sent for histopathological assessment to the Pathology and Laboratory Medicine Department at King Saud University Medical City from 2015 to 2019. The study proposal was reviewed by the HEC/IRB board at our institution and was granted approval on January 14, 2020. The patients’ basic demographic data, clinical diagnosis, and histopathological diagnosis were collected using a data collection form. The demographics and clinical information were collected through chart review and entered into an Excel sheet. The histopathological slides were reviewed by a single pathologist for confirmation of the diagnosis and for categorization.

All specimens were routinely processed with the preparation of hematoxylin and eosin-stained slides and additional special stains and/or immunohistochemical (IHC) stains whenever applicable. The categorization of the clinical diagnoses was adopted from the World Health Organization (WHO) classification of Tumors of the Eye [8] with corresponding matching histopathological diagnoses for easier final data analysis as follows: fibrodegenerative/proliferative, melanocytic, inflammatory, epithelial lesions, hematolymphoid, tumor-like lesions, hamartoma/choristoma, and vascular lesions. Data were also compared between the two main groups of benign entities and malignant lesions. We also compared the prevalence and frequency of lesions between adults and children (16 years of age or younger). Finally, we added a brief description and more discussion on the most encountered type(s) of lesions in each age group.

### Statistical Analysis

Data were collected, managed, and coded in a spreadsheet using Microsoft Excel 2010® software. They were analyzed using SPSS® version 21.0 (IBM Inc., Chicago, Illinois, USA).

Descriptive analysis was performed, where categorical variables were presented in the form of frequencies and percentages. Data exploration using the Shapiro–Wilk test was performed for the continuous variables; the data were found to be not normally distributed and, hence, are presented as the median (interquartile range) (IQR). However, the mean age was also calculated and used in the discussion for comparison with other similar studies whenever applicable. Consequently, the Mann–Whitney test was used for comparisons between any two groups for the continuous variables, and the chi-squared test was used for comparing proportions between the groups. Any output with a *p* value below 0.05 was interpreted as an indicator of statistical significance.

## Results

**Fig. 1:**
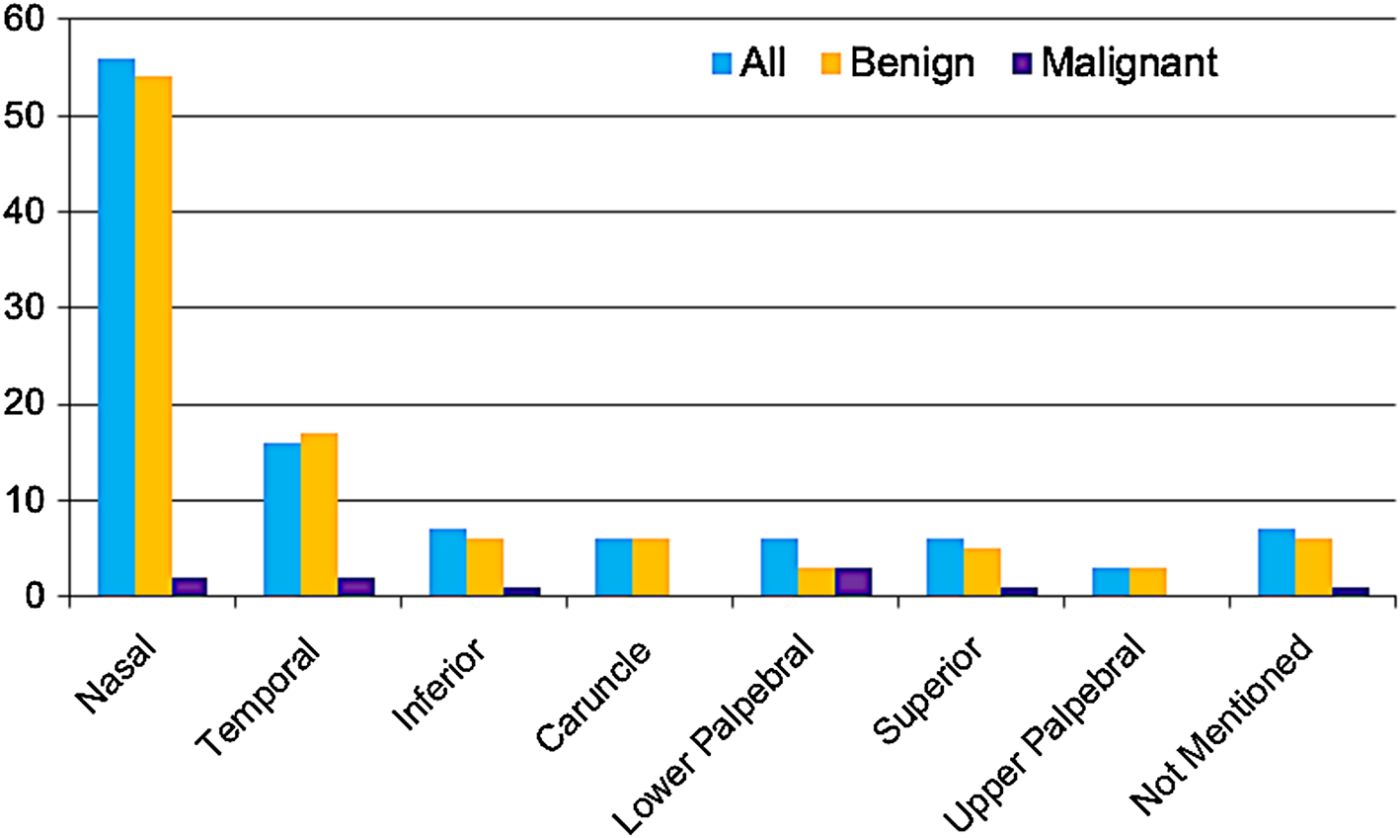
Distribution of 110 benign and malignant conjunctival lesions based on location

A total of 110 conjunctival specimens from 108 patients were received for histopathological examination within the review period. The mean age of patients at presentation was 46.4 ± 22.9 years (range 1 month – 84 years, with a median of 53.0 (IQR: 27.0–66.0)), and most patients were adults (83.6%). Sixty-seven patients were males (60.9%), and 43 were females (39.1%). The median duration of symptoms for all lesions collectively was 1.8 months (IQR: 0.2–11.9 months), with a significantly shorter duration for malignant lesions (median 0.6 compared to 2.1 months) than for benign lesions (*p* = 0.036). The median follow-up duration was 2.8 months. The majority of lesions were benign (91%), and these were mostly found in the nasal aspect of the conjunctiva (54%). In contrast, the most common location of malignant lesions was the lower palpebral conjunctiva (Fig. 1). The lesions involving the caruncle were all benign and included two nevi (1 compound and 1 subepithelial nevus), two benign reactive lymphoid hyperplasia (BRLH), one trichilemmal cyst, and one nonspecific chronic inflammation. The patients’ demographics and characteristics are summarized in Table 1. The final histopathological diagnosis was compared to the clinical diagnosis and found to be matching in 75.5% of the total specimens; however, the frequency of accurately matching diagnosis was higher among the benign lesions than the malignant entities (77% compared to 60%), noting that some specimens were labeled as unspecified masses/lesions that lowered this percentage.

**Table 1:** Summary of the demographic data and other basic characteristics among benign versus malignant 110 conjunctival lesions

Characteristic	All (*n* = 110) *n* (%)	Benign (*n* = 100) *n* (%)	Malignant (*n* = 10) *n* (%)	*p* value
Age of presentation (years) Median (IQR)	53.0 (27.0–66.0)	52.5 (26.0–65.8)	53.0 (43.5–74.0)	0.120
*Gender*				
Male	67 (60.9)	62 (62.0)	5 (50.0)	0.458
Female	43 (39.1)	38 (38.0)	5 (50.0)	
Age at surgery (years) Median (IQR)	53.0 (27.0–66.5)	52.5 (26.0–66.0)	53.0 (43.5–74.8)	0.318
*Age group*				
Children (≤ 16 years)	18 (16.4)	18 (18.0)	0 (0.0)	0.142
Adults (> 16 years)	92 (83.6)	82 (82.0)	10 (100)	
*Bilateral*				
Yes	17 (15.5)	17 (17.0)	0 (0.0)	0.156
No	93 (84.5)	83 (83.0)	10 (100)	
Duration of symptoms (months) Median (IQR)	1.8 (0.2–11.9)	2.1 (0.2–12.3)	0.6 (0.06–6.4)	0.036*
Duration of Follow-up (months) Median (IQR)	2.8 (0.5–13.7)	2.9 (0.6–14.3)	1.3 (0.03–6.5)	0.261
*Clinical diagnosis matching* the histopathological diagnosis				
Yes	83 (75.5)	77 (77.0)	6 (60.0)	NA
No	27 (24.5)	23 (23.0)	4 (40.0)	NA

*Statistically significant at 5% level of significance

**Fig. 2:**
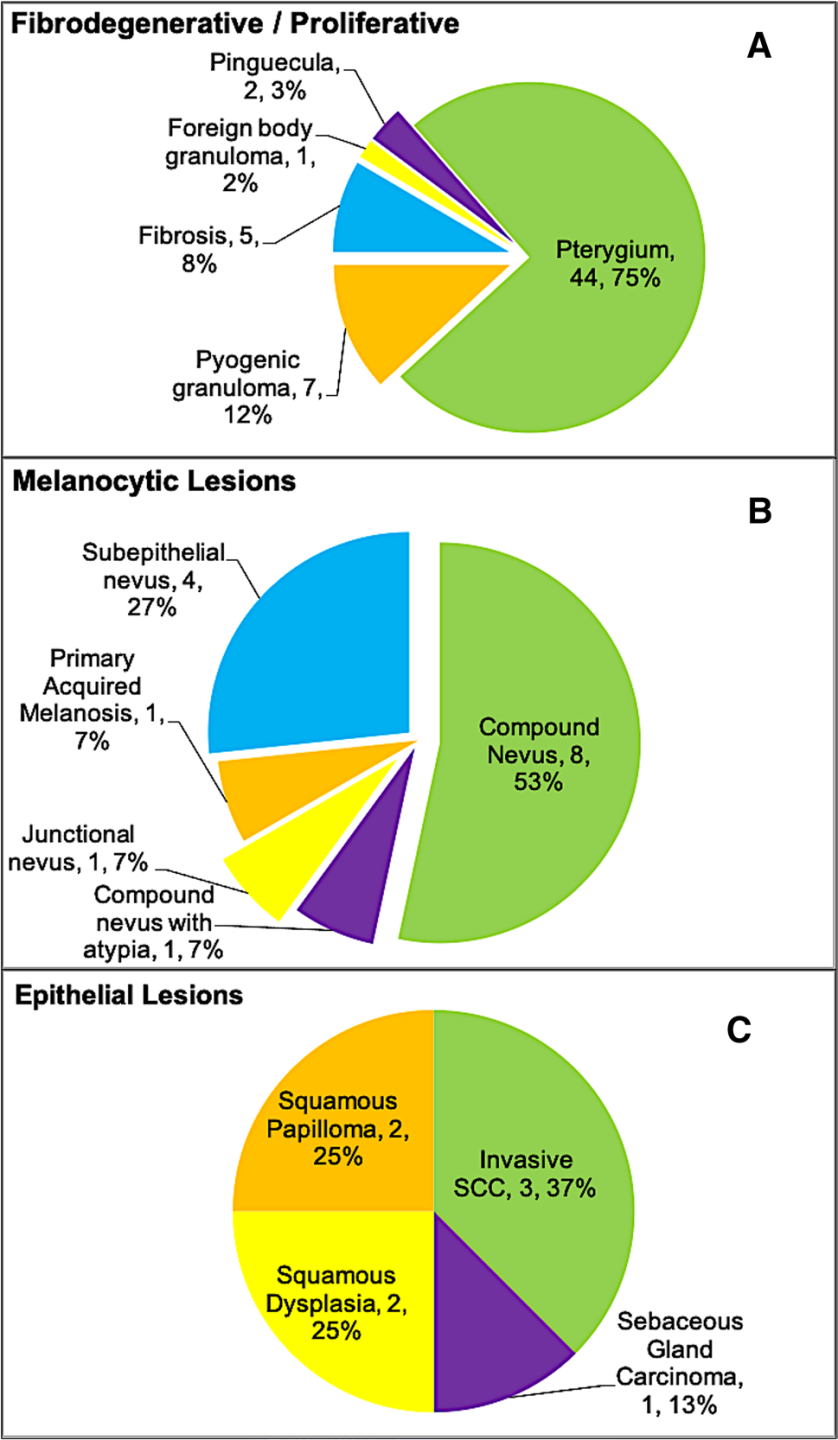
Distribution of the most common conjunctival lesions in adults based on their final histopathological diagnosis according to the WHO categorization of diagnoses with demonstration of subtypes in each entity or category. **A** Fibrodegenerative and proliferative, **B** Melanocytic, and **C** Epithelial

The most frequent category of lesions was fibrodegenerative and proliferative lesions in 53.6%, with the majority being either pterygia or pinguecula (46/59), followed by melanocytic lesions in 13.6%, with the majority being benign compound conjunctival nevi (8/14). These are demonstrated in Table 2 with a detailed distribution of the sub entities in each of these two most common categories in Fig. 2A & B. The most frequent malignant lesions were mostly epithelial (6/10), with OSSN being the most common (five cases), in addition to one case of sebaceous gland carcinoma, as shown in Fig. 2C. When the same categories were analyzed according to gender, the fibrodegenerative/proliferative lesions were found mostly among males 42/59 (71.2%) versus 17/59 females (28.8%), with a statistically significant *P* value (*p* < 0.001*). There was no statistically significant difference in the distribution of other categories between males and females (Table 2).

**Table 2:** Distribution of benign and malignant entities in 110 conjunctival lesions as per the WHO categorization based on gender and the age group

WHO lesion category	All (*n* = 110) *n* (%)	Gender		Benign (*n* = 100)		Malignant (*n* = 10)	
		Males (*n* = 67) *n* (%)	Females (*n* = 43) *n* (%)	Children ≤ 16 y (Total *n* = 18) *n* (%)	Adults > 16 y (Total *n* = 92) *n* (%)	Children ≤ 16 y (Total *n* = 18) *n* (%)	Adults > 16 y (Total *n* = 92) *n* (%)
Fibro-degenerative / Proliferative	59 (53.6)	42 (62.7)	17 (39.5) (42/59 (71.2%) vs. 17/59 (28.8%) *p* < 0.001*)	3 (16.7)	56 (60.9)	0 (0.0)	0 (0.0)
Melanocytic Lesions	15 (13.6)	7 (10.4)	8 (18.6)	6 (33.3)	8 (8.7)	0 (0.0)	1 (1.1)
Inflammatory	10 (9.1)	5 (7.5)	5 (11.6)	0 (0.0)	10 (10.9)	NA	NA
Epithelial Lesions	8 (7.3)	6 (9.0)	2 (4.7)	0 (0.0)	2 (2.0)	0 (0.0)	6 (6.5)
Hematolymphoid Lesions	7 (6.4)	3 (4.5)	4 (9.3)	3 (16.7)	0 (0.0)	0 (0.0)	4 (4.3)
Tumor-like lesions	6 (5.5)	3 (4.5)	3 (7.0)	3 (16.7)	3 (3.3)	NA	NA
Hamartoma & Choristoma	3 (2.7)	1 (1.5)	2 (4.7)	3 (16.7)	0 (0.0)	NA	NA
Vascular Lesions	2 (1.8)	0 (0.0)	2 (4.7)	0 (0.0)	2 (2.2)	0 (0.0)	0 (0.0)

*WHO* World Health Organization

*Statistically significant at 5% level of significance

*NA* not applicable; *Y* years

**Fig. 3:**
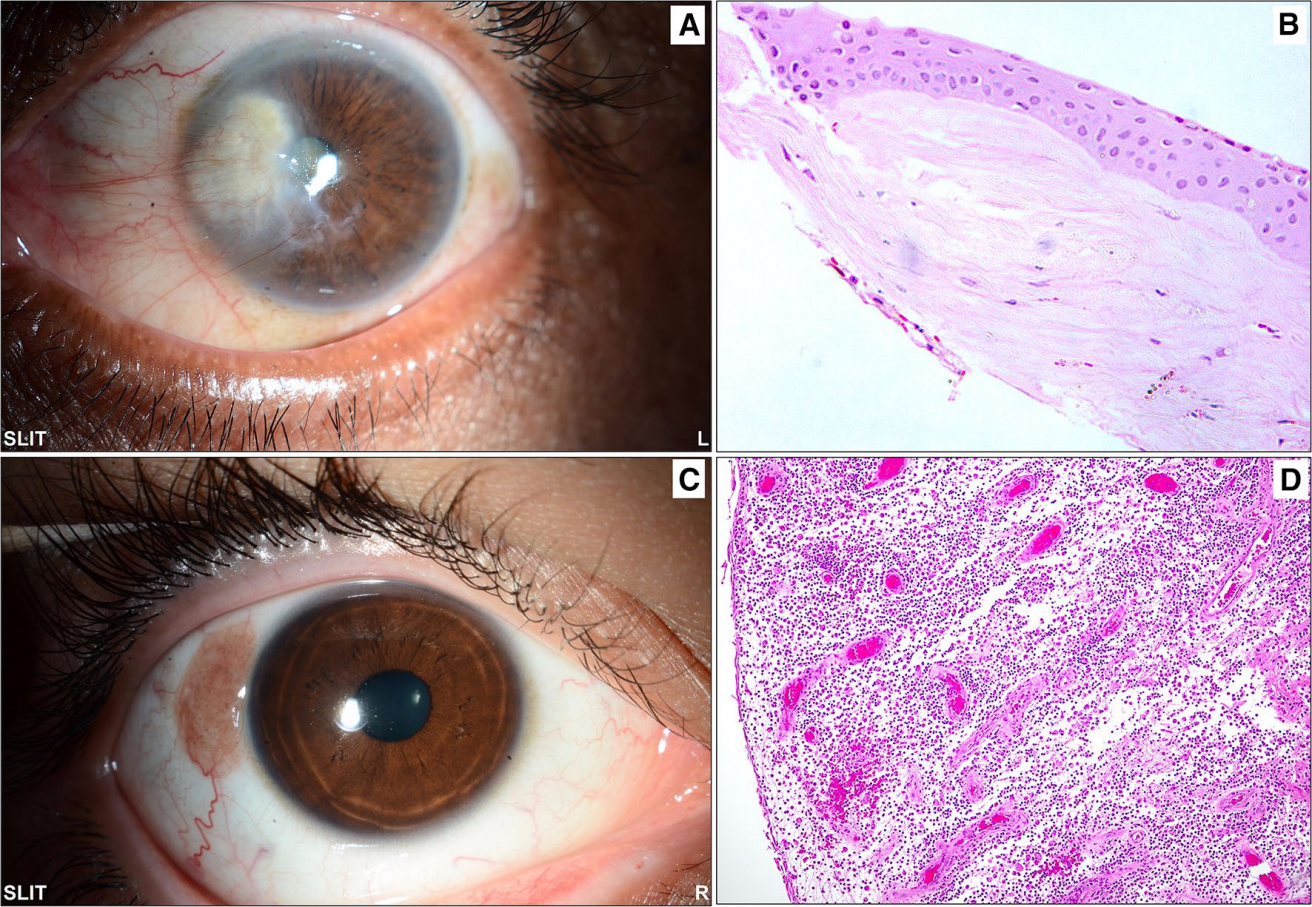
**A** The clinical appearance of an advancing nasal pterygium, which was the most commonly excised lesion in adults approaching the cornea and interfering with the visual axis. **B** The corresponding histopathological appearance of the shaved corneal end of the pterygium with irregular epithelium and scarring of the anterior stroma (original magnification × 200 hematoxylin and eosin). **C** A sessile gelatinous temporal conjunctival lesion in a 16-year-old male that was excised and sent for histopathological examination with a nonspecific clinical diagnosis of a “mass”. **D** The histopathology of pyogenic granulomas showing proliferating capillaries within loose stroma (original magnification × 100 hematoxylin and eosin)

**Fig. 4:**
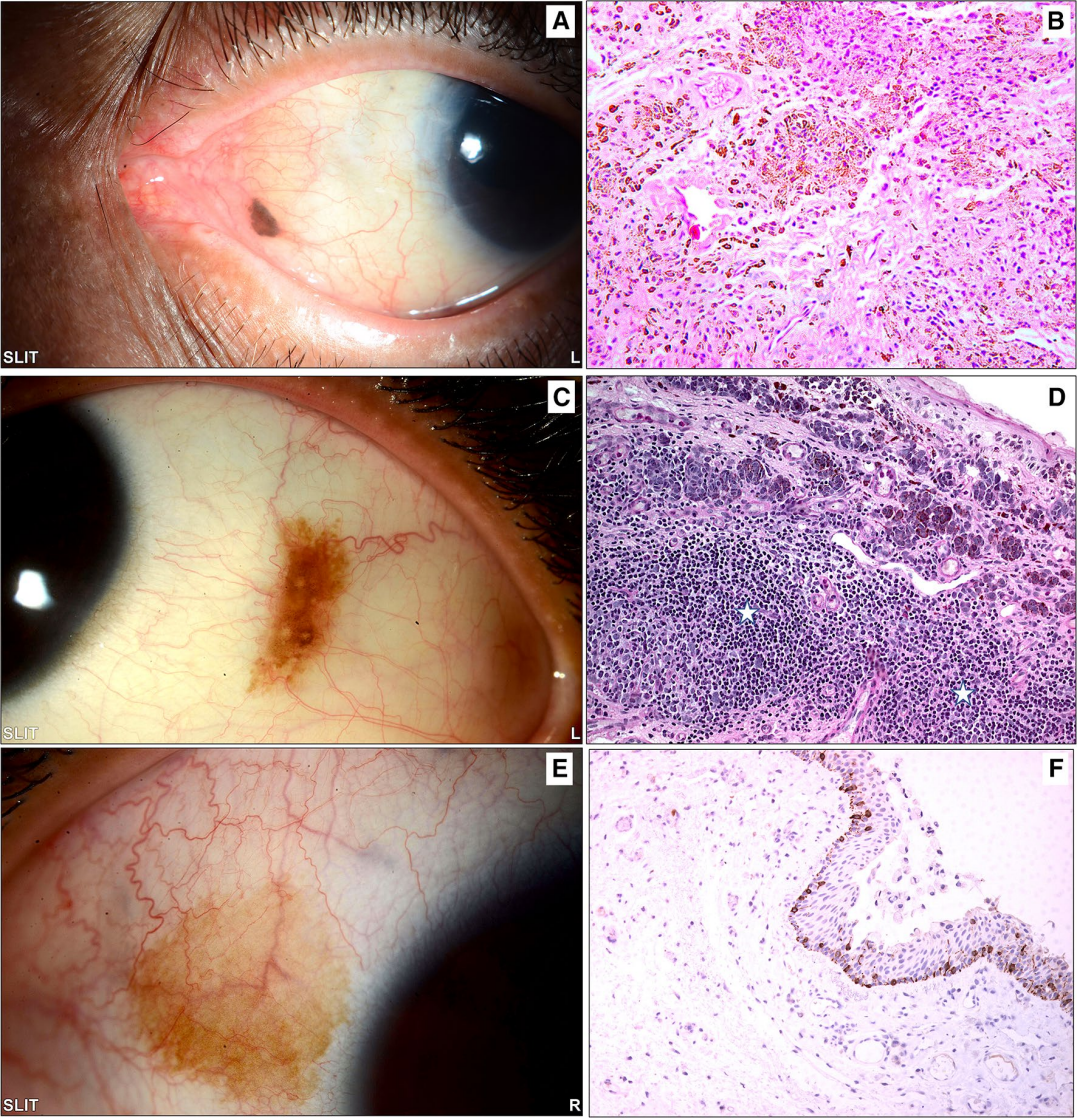
**A** The clinical photo of a small left eye nasal conjunctival pigmented lesion in an adult treated by excisional biopsy. **B** The histopathology of the same lesion showing melanocytic cells within the stroma with mild architectural atypia (loss of maturity) and no cytological atypia, finally diagnosed as a benign subepithelial nevus (original magnification × 200 hematoxylin and eosin). **C** The clinical appearance of a left eye temporal conjunctival pigmented lesion in a 15-year-old girl treated by excisional biopsy because of recent growth. **D** An example of the histopathological appearance of a conjunctival inflamed juvenile compound nevus (IJCN) with stromal lymphocytic infiltration (white stars) causing an increase in the nevus size clinically (original magnification × 200 periodic acid Schiff). **E** The clinical appearance of a lightly pigmented patch in a 30-year-old female that was excised without specific clinical diagnosis. **F** The histopathology of the conjunctival lesion revealed melanocytic hyperplasia without atypia, and the melanocytes at the base of the conjunctival epithelium expressed reactivity to the diagnostic immunohistochemical marker (original magnification × 100 Melan-A)

**Fig. 5:**
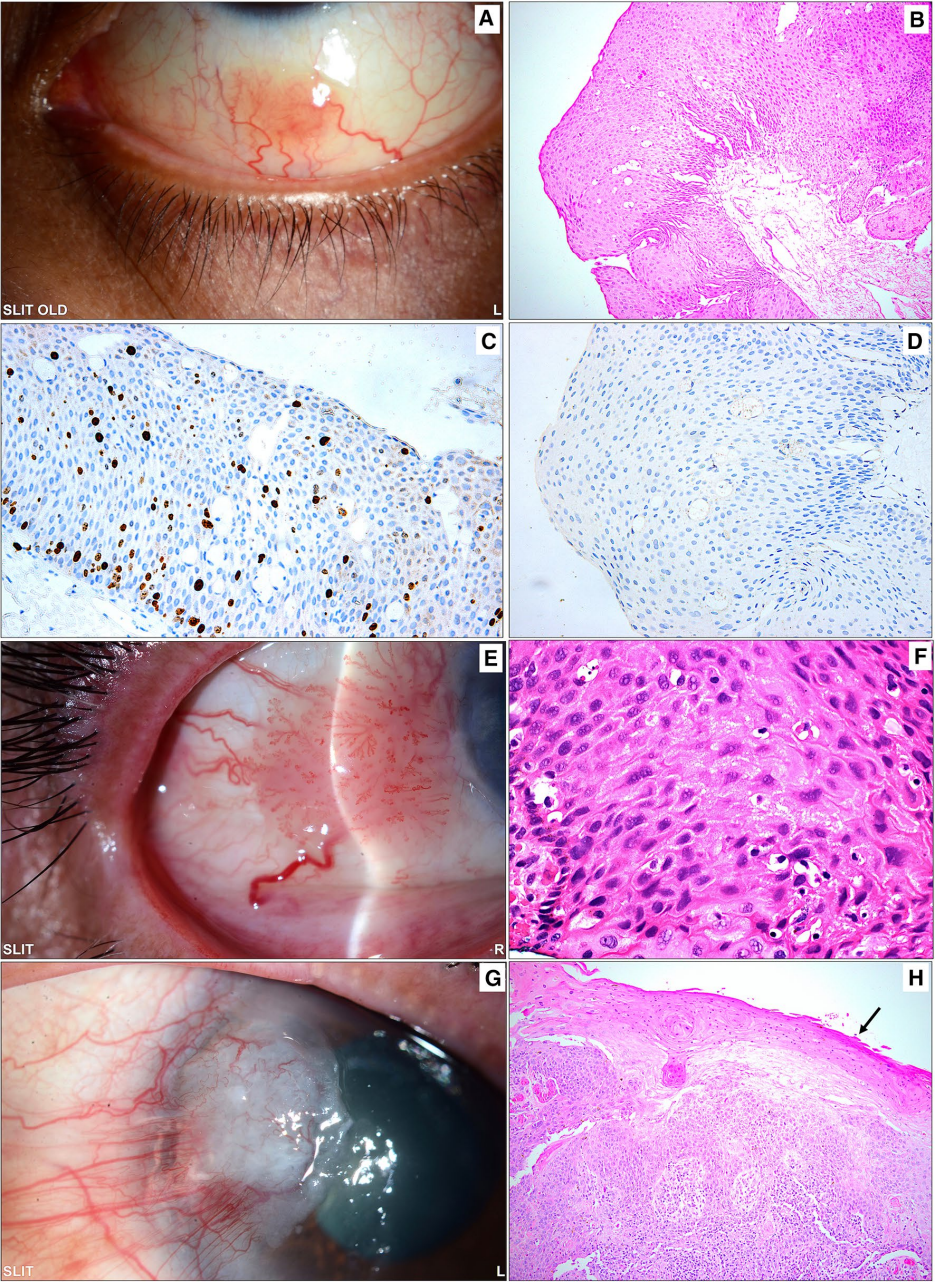
**A** Sessile gelatinous inferior conjunctival lesion in an adult female (33 years old) diagnosed clinically as a squamous papilloma. **B** The corresponding histopathological appearance of the papillomatous lesion with thick epithelium, absent dysplasia, and underlying fibrovascular core (original magnification × 200 hematoxylin and eosin). **C** The same lesion above showing a moderate proliferation index (original magnification × 200 Ki67). **D** The cells in the lesion did not show any reaction to human papilloma virus (HPV) antibodies, and a verrucous papilloma was excluded in this case (original magnification × 200 HPV). **E** Another sessile papillomatous temporal conjunctival lesion with a feeder blood vessel in a 72-year-old male treated by excisional biopsy with a clinical diagnosis of suspected OSSN. **F** The histopathological examination of a conjunctival lesion showing thick epithelium with moderate-to-severe dysplasia not involving the full thickness of the epithelium (original magnification × 400 hematoxylin and eosin). **G** The clinical appearance of a pterygium-like lesion extending into the cornea and showing evidence of leucoplakia. **H** Excised tissue showing histopathological evidence of invasive squamous cell carcinoma (SCC) with overlying metaplasia of the conjunctival epithelium and early keratinization (black arrow) corresponding to the area of leukoplakia (original magnification × 100 hematoxylin and eosin). Note the overlapping clinical appearance between a normal pterygium in Fig. 3A and the pterygium-like SCC in this latest case and the similarity of the sessile lesions in **A** and **E** that might lead to clinical misdiagnosis

**Fig. 6:**
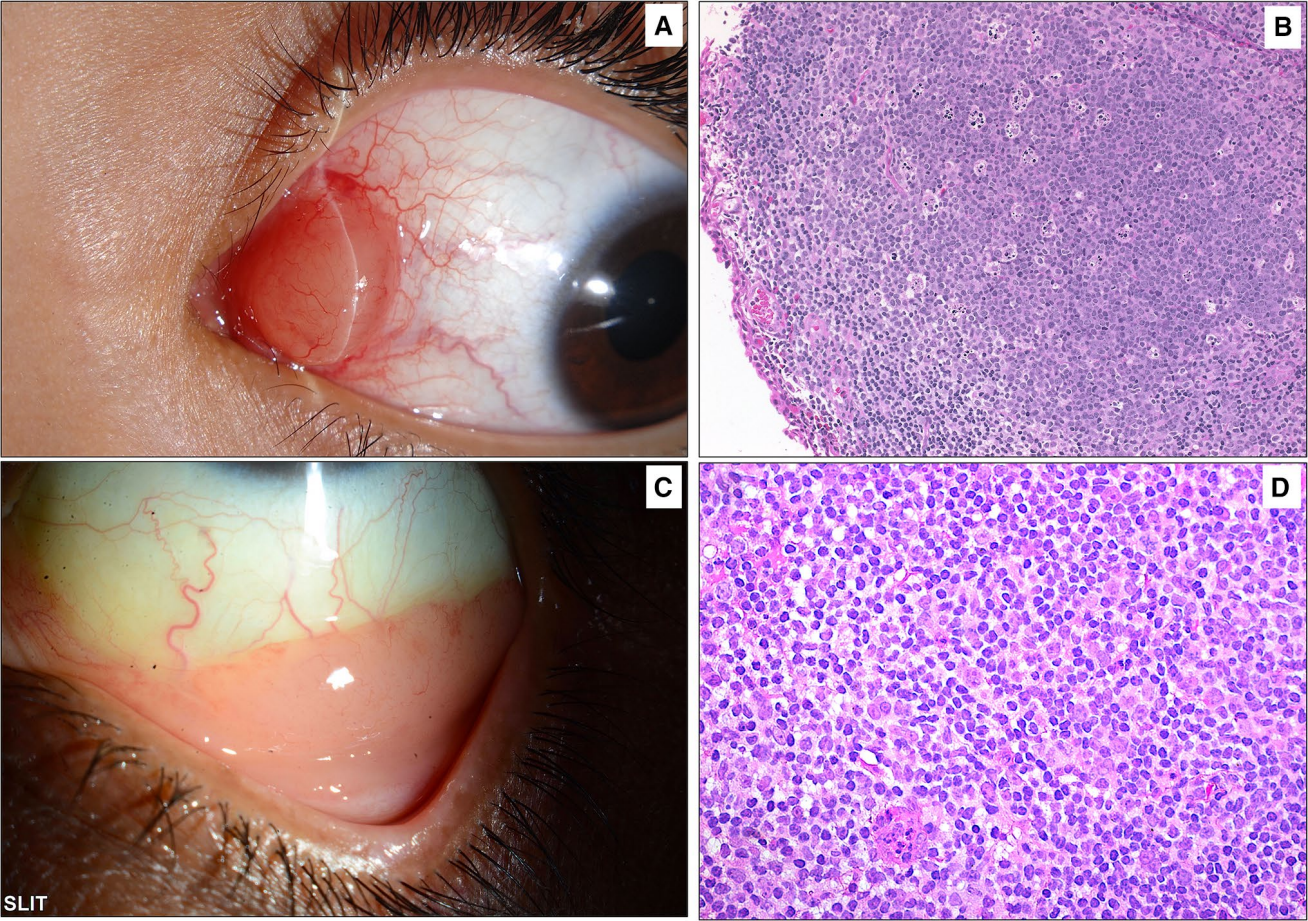
**A** Clinical example of a pinkish conjunctival mass located in the caruncle of a child. **B** The histopathology of the lesion showing subepithelial mature lymphocytic infiltrate with follicle formation and germinal centers. The lesion was proven to represent benign reactive lymphoid hyperplasia based on the immunohistochemical staining results (original magnification × 200 hematoxylin and eosin). **C** The typical salmon patch involving the lower conjunctival fornix in a 53-year-old female with a previous history of autoimmune hepatitis. The lesion was surgically excised. **D** The histopathology of the sheets of mature lymphocytes representing mucosal-associated lymphoid tumor (MALT) lymphoma (original magnification × 400 hematoxylin and eosin)

**Fig. 7:**
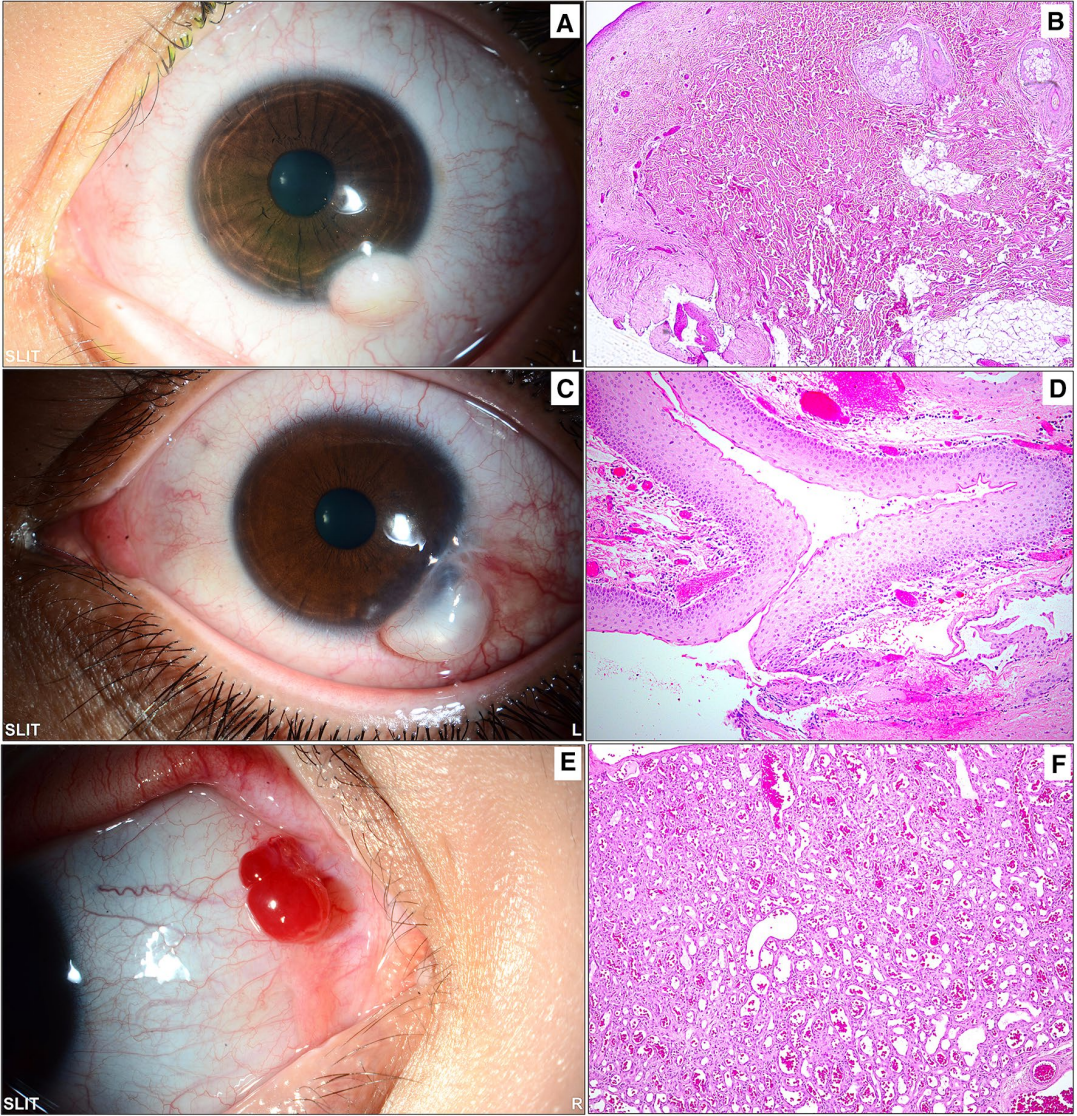
Miscellaneous lesions: **A** A whitish inferior dome-shaped limbal mass in a child diagnosed clinically as limbal dermoid. **B** The same lesion following excisional biopsy showing wavy thick collagenous fibers and fat with overlying skin-like epithelium and pilosebaceous units, confirming the diagnosis of a choristoma (original magnification × 50 hematoxylin and eosin). **C** The clinical appearance of a tumor-like cystic lesion in a 21-year-old male with long-standing severe vernal keratoconjunctivitis. **D** Histopathological picture of the collapsed cyst wall following excisional biopsy. The wall consists of nonkeratinizing stratified squamous epithelium representing the wall of an inclusion cyst (original magnification × 100 hematoxylin and eosin). **E** An example of a partially cystic red conjunctival lesion in a young female that was thought to be vascular in clinical nature. **F** The histopathology image showing lobular areas of capillary proliferation with the final diagnosis of a capillary hemangioma (original magnification × 100 hematoxylin and eosin)

According to age, the most common lesions among adults in order of frequency were fibrodegenerative/proliferative (60.9%), followed by inflammatory lesions (10.9%), melanocytic lesions (9.8%), and epithelial lesions (8.7%). Other less frequent lesions included hematolymphoid, tumor-like, and vascular lesions (4.3%, 3.3%, and 2.2%, respectively). No developmental lesions (hamartoma/choristoma) were encountered in adults. In contrast, melanocytic lesions were more commonly observed among children in 6/18 lesions (33.3%). The rest of the lesions were equally divided (three lesions each) among the following categories: fibrodegenerative, hematolymphoid, tumor-like lesions (cysts), and hamartoma/choristoma (Table 2). We further analyzed the above four most common categories in adults (fibrodegenerative/proliferative, inflammatory, melanocytic, and epithelial) looking at the following variables: age at presentation, gender, duration of symptoms, bilaterality, and outcome. The mean age for melanocytic lesions was younger (36. ± 17.3 years) compared to the remaining three categories, with a statistically significant difference (*P* = 0.013*). The median duration of symptoms was not found to be significantly different among the four groups (*P* = *0.086*). There was no statistically significant difference comparing the rest of the variables above between the four categories; however, the favorable outcome was observed to be the least common in 62.5% of the epithelial lesions (*P* = *0.333),* possibly because 75% of these lesions were either malignant or premalignant (dysplasia). Furthermore, most of the lesions in the four major categories were unilateral (81.9%). Bilateral lesions were found in inflammatory lesions (in 40%), which was expected, followed by fibrodegenerative/proliferative lesions (in 19.6%), while melanocytic and epithelial lesions were unilateral in all adult patients (Table 3). The other categories each had a small number of lesions and were not suitable for further analysis. Melanocytic lesions, which were the most frequent category in the pediatric age group, were all benign conjunctival nevi. Clinicopathological examples of the important diagnoses in the above categories are demonstrated in Figs. 3, 4, 5, 6 and 7.

**Table 3:** Comparison of the 4 commonest categories of lesions in adults regarding age, duration of symptoms, gender, bilaterality, and outcome

Characteristic	Total (*n* = 83) *n* (%)	Fibro-degenerative / Proliferative (*n* = 56) *n* (%)	Melanocytic lesions (*n* = 9) *n* (%)	Inflammatory (*n* = 10) *n* (%)	Epithelial lesions (*n* = 8) *n* (%)
Age at presentation (years) Mean ± SD [Range]	54.4 ± 17.7 [18.0–84.0]	56.0 ± 16.3 [20.0–84.0]	36.7 ± 17.3 [18.0–72.0]	58.6 ± 17.8 [24.0–78.0]	57.8 ± 18.6 [33.0–83.0] *p* = 0.013*
Duration of symptoms (months) Median (IQR)	2.1 (0.2–11.0)	3.9 (0.3–15.2)	0.5 (0.1–11.0)	0.5 (0.1–3.6)	1.2 (0.1–4.4) *p* = 0.086
*Gender*					
Male	54 (65.1)	40 (71.4)	3 (33.3)	5 (50.0)	6 (75.0) *p* = 0.097
Female	29 (34.9)	16 (28.6)	6 (66.7)	5 (50.0)	2 (25.0)
*Bilaterality*					
Yes	15 (18.1)	11 (19.6)	0 (0.0)	4 (40.0)	0 (0.0) *p* = 0.069
No	68 (81.9)	45 (80.4)	9 (100)	6 (60.0)	8 (100)
Duration of Follow-up (months) Median (IQR)	3.6 (0.8–15.1)	3.4 (0.9–17.8)	0.7 (0.3–8.8)	5.3 (1.6–17.1)	6.9 (1.1–22.7)
*Outcome*					
Favorable	66 (79.5)	45 (80.4)	7 (77.8)	9 (90.0)	5 (62.5) *p* = *0.333*
Unfavorable	8 (9.6)	6 (10.7)	0 (0.0)	1 (10.0)	1 (12.5)
No FU	9 (10.8)	5 (8.9)	2 (22.2)	(0.0)	2 (25.0)

*Statistically significant at 5% level of significance

**Fig. 8:**
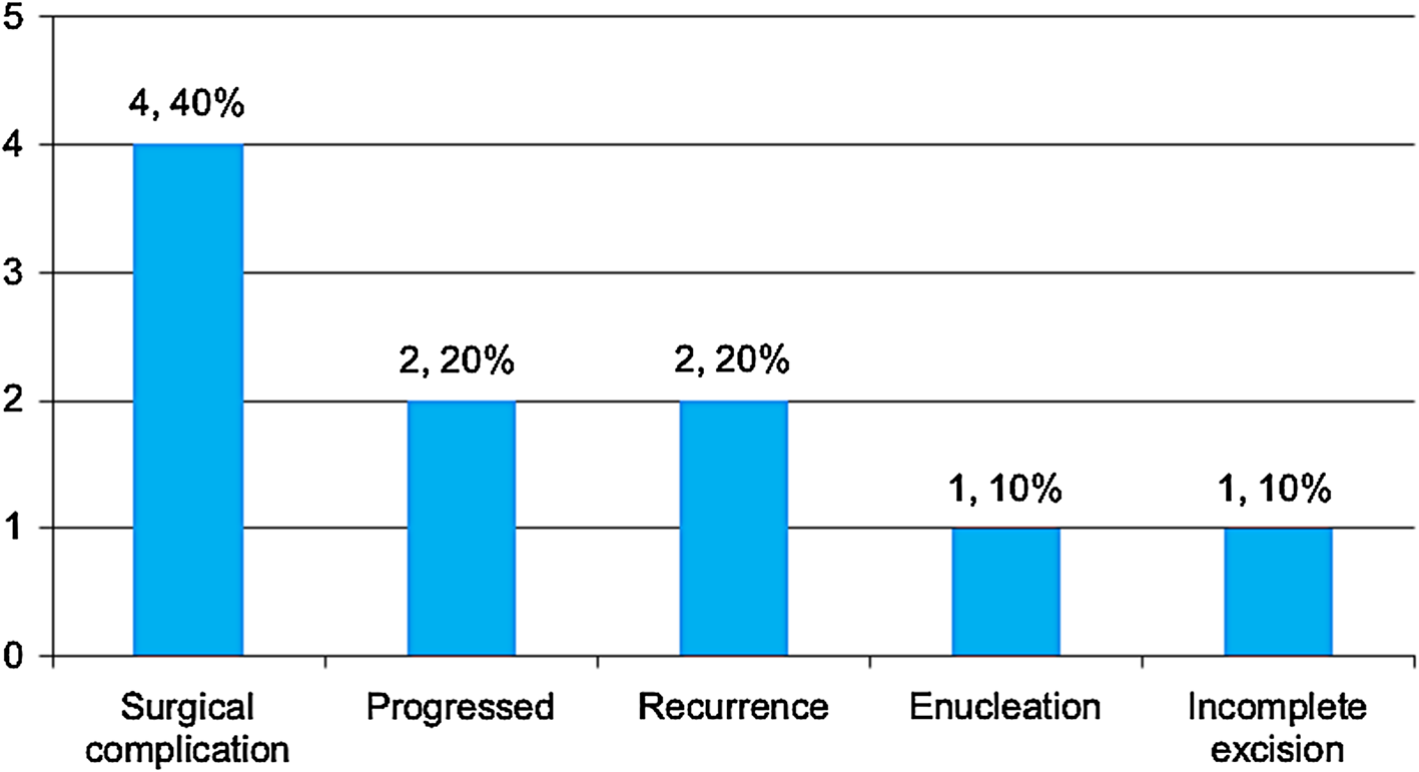
Demonstration of different reasons to label the outcome in 10 conjunctival lesions as being unfavorable

The outcome of all lesions was favorable in 84 patients (76.4%) and unfavorable in 10 patients (9.1%), whereas the remaining 16 (14.5%) were lost to follow-up. When the outcome was calculated only for lesions with follow-up information, an overall favorable outcome was observed in 89.4% (84/94) of the lesions. Figure 8 demonstrates the details of the unfavorable outcomes among the remaining 10 lesions.

## Discussion

Conjunctival lesions are common with a wide spectrum of benign, premalignant, and malignant lesions. The clinicopathological characteristics of conjunctival lesions have been studied in the literature with variable study designs and different classifications of these lesions because of their diversity. In our study, we elected to follow a classification that was adopted from the latest WHO classification of tumors of the eye with an emphasis on comparison between benign and malignant lesions in adults versus children (defined in our center as 16 years of age or younger) [8].

The median age of patients at presentation in our study was 53.0 (IQR 27.0–66.0). There has been wide variation in the age presentation of conjunctival lesions in different studies, with a mean ranging from 27.7 years to 59.9 years [1, 2, 9, 10]. In our study, the mean age was within the same range (46.45 years). Of our 110 cases, 18 lesions were found in children aged ≤ 16 years (16.4%), and 92 lesions were found in adults aged > 16 years (83.6%). Most studies did not find a significant sex predilection between males and females [1, 2, 10]. However, although not statistically significant, males were more commonly affected in all studies, including ours, except for Chi and his coauthor, where the ratio was approximately reversed, and Aliakbar-Navahi et al., who reported a male-to-female ratio that was almost equal [1, 2, 10, 11]. The authors in the latter attributed this finding to the fact that females in Iran were more likely to spend time outdoors [11]. The closest male-to-female ratio to ours was reported by Alves et al., who found a higher incidence of lesions in males (69% compared to our 60.9%) than in females (31% compared to our 39.1%) [9].

Benign lesions were exceedingly more common than malignant lesions in our cohort (91% benign versus 9% malignant). The most common overall lesions among both categories of lesions were fibrodegenerative lesions (53.6%), followed by melanocytic lesions (13.6%). This result is quite similar to what Khan found (55.03% and 12.40%, respectively) [12]. Similarly, Aliakbar-Navahi et al. reported that the most common lesion among all their cases was pterygia, accounting for 69.2%, followed by melanocytic lesions, accounting for 15.3% [11]. However, in Iran, when the study design was focused only on neoplastic lesions of the conjunctiva, melanocytic lesions (nevi) ranked first as the most common primary neoplastic lesion in more than one-third of the cases, followed by SCC [13]. In our study, all the malignant lesions were strictly unilateral, contrary to the benign lesions, which were bilateral in 17% of cases.

Most of the cases in Khan et al.’s study involved limbal and perilimbal lesions in 71.10% and bulbar lesions in 3.13% [12]. In our study, 68.2% of all lesions involved the bulbar conjunctiva nasally and temporally, whereas the superior and inferior conjunctiva were involved in 11.9%. As the exact locations of limbal and perilimbal lesions were not defined in our data, we were only able to compare the collective bulbar conjunctival lesions in all four quadrants (80.1%) to Khan’s combined limbal, perilimbal, and bulbar lesions (74.23%), which were almost similar [12]. This location is of particular importance in regard to the analysis of benign lesions alone because almost all the nasal bulbar conjunctival lesions in our study were benign (54 out of 56 nasal lesions), whereas the most common location of malignant lesions was the lower palpebral conjunctiva. This was also pointed out in some reports [4]. Regarding the unique location of lesions in the caruncle, Solari et al. found that the majority of these lesions in 42 cases were epithelial (38.09%), inflammatory (31.7%), and melanocytic (21.95%) in order of frequency, whereas the Shields group found a higher prevalence of squamous papilloma (32%), followed by nevi (24%) [14, 15]. In our study, the cases were mostly melanocytic or BRLH. The accuracy of the preoperative clinical diagnosis (confirmed histopathologically) for the lesions of the caruncle was also noted to range only from about 50% to 60.71% in their cases [14, 16].

When comparing the prevalence of benign lesions in our two age groups, all 18 lesions in children ≤ 16 years old were found to be benign, while 82/92 lesions in adults were benign. The most common lesions in children were melanocytic lesions in 6/18 cases (33.3%), similar to previous reports [4], all of which were compound nevi (1/6 with atypia). It is also worth mentioning that in terms of age, no epithelial or inflammatory lesions or pterygia were seen in children. Melanocytic lesions in the form of nevi tend to show some growth related to puberty and/or inflammation; hence, those lesions are usually removed at adolescence [12, 17, 18]. However, in adults, malignant transformation has been found to be very low and does not warrant excision solely to avoid this risk [19]. In our study, the nevi that were excised in adults were mainly removed for cosmetic reasons. Hematolymphoid lesions in children accounted for 16.7%, which were all benign reactive lymphoid hyperplasia (BRLH), compared to adults, where they accounted for 4.3% only and were all malignant lymphomas involving the lower palpebral conjunctiva. Shields et al. studied conjunctival lymphoproliferative lesions and reported that lymphoma rather than BRLH is more likely to occur with higher median age and fornix location [7]. Another observation is the category of hamartomas & choristomas, which was only found in children in 16.7% of cases, with no cases in adults, as would be expected.

We categorized the lesions according to the WHO book on tumors of the eye classification, and the most common benign lesions encountered were fibrodegenerative lesions (59%), followed by melanocytic lesions (14%) and inflammatory lesions (10%). In Chi and Beak, the most common category was melanocytic lesions, accounting for 54.2%, most of which were compound nevi [10]. In our fibrodegenerative category, most lesions were pterygia/pinguecula (44/59 lesions). Similarly, Findik reported that among their nonneoplastic lesions, 54.7% were pterygia [2]. This variation is most likely related to ethnic, racial, and geographical (environmental) factors. This was further clarified in our study when we analyzed the frequency of the fibrodegenerative category according to gender. This type of lesion was found to be more prevalent in males than in females, with a statistically significant *p* value of < 0.001, which was not found in any other category. Other studies have also reported a high prevalence of pterygia, with an unusually higher proportion in women than men [2, 20]. We attribute this to climatic and environmental factors related to heat and sand in addition to our cultural lifestyle, where females tend to spend more time indoors, avoiding the risk factors for pterygia, contrary to the observed different lifestyles of women in Iran. In adults, we also observed that fibrodegenerative and inflammatory conjunctival lesions might be observed bilaterally in 19.6% and 40% of patients, respectively, whereas melanocytic and epithelial lesions were strictly unilateral.

The prevalence of malignant conjunctival lesions in our study was 9%, comparable to the overall prevalence of malignant conjunctival lesions reported in previous studies [10, 11, 21]. In the current study, the most frequent malignant lesion was ocular surface squamous neoplasia (OSSN), which was observed in 5/10 adult specimens (50%), with no gender predilection; this finding is consistent with the findings of Elshazly and Aliakbar-Navahi et al. [1, 11], despite previous reports of a higher incidence of OSSN in males [7, 22–24]. The differences in the spectrum of OSSN could be attributed to racial and environmental factors, which was further illustrated in a study by Gichuhi et al. describing the spectrum of OSSN in Africa, where it was nearly equally common in women and men [25]. On the other hand, others who limited their study to neoplastic lesions alone reported SCC as the most common epithelial conjunctival tumor, with an almost double prevalence in males (65.3%) due to their lifestyle in Iran [23]. In another broader study on eye tumors in a large referral eye center in our area by Huaman and Cavender, SCC (mostly in the bulbar followed by the limbal conjunctiva) was the second most common ocular tumor, with almost double prevalence among males (62%) and an average age for conjunctival intraepithelial neoplasm (CIN) of 51.4 years [26]. There might have been a gradual change in lifestyle in our country, with more involvement of females in outdoor work and/or activities since the time of that study in 1991 and a corresponding shift in the gender-based equal prevalence observed recently in our current study. We also observed a wide spectrum of intraepithelial diseases of the conjunctiva, as clearly described by Mudhar in his review. We had cases ranging from mild squamous dysplasia to severe dysplasia, often with papillomatous configuration (with IHC staining for human papilloma virus), as we have demonstrated in Fig. 5, to invasive SCC [18].

The second most common lesion within our study was extranodal marginal zone lymphoma (ENMZL), previously termed mucosal-associated lymphoid tissue (MALT), observed in 3/10 specimens (20%), similar to previous studies [2].

Conjunctival tumors are usually recognized by their behavior and clinical appearance, although there are some diagnostic challenges that require histopathological evaluation [10]. In our cases, as demonstrated in the clinicopathological Figs.3, 4, 5, 6, the diagnosis was not accurately suspected in all cases because of a few mimickers. Therefore, careful histopathological examination in all cases, especially for OSSN, is essential for diagnostic purposes [27]. The overall rate of precision in clinical diagnosis in the current study was 75.5%, corresponding to equivalent or even higher rates in other similar studies [11, 14, 28, 29]. This number, however, might be an underestimate, as some specimens were labeled “mass” or “lesion” without a specific clinical diagnosis, which might have affected this percentage. Ostergaard et al. reported a higher frequency of matching clinical diagnosis within the malignant lesion group [28]; however, our data showed that the accurate matching diagnosis frequency was better among benign lesions than among malignant entities (77% compared to 60%).

All our lesions were managed properly, mostly by excisional biopsy in 93.6%, whereas incisional biopsy was performed for the diagnostic purpose of 6 benign inflammatory conditions (mostly ocular cicatricial pemphigoid) and one case of MALT lymphoma. As previously mentioned, due to the variety of study designs and approaches to addressing conjunctival lesions, the management and outcome of these lesions have been inconsistently reported, depending on the type of lesions. Within our study, the overall outcome was favorable in 84 patients (76.4%). Upon further analysis of the four most common entities in adults (fibrodegenerative/proliferative, inflammatory, melanocytic, and epithelial), a favorable outcome was observed to be the least common in epithelial lesions (80.4%, 90%, 77.8%, and 62.5%, respectively). This low percentage could be explained by the fact that the majority of the epithelial lesions are either malignant or dysplastic at presentation. An unfavorable outcome was recorded in only 10 patients, whereas the remaining 16 patients were lost to follow-up. Most of the unfavorable outcomes were related to surgical complications in 4/10 (scleral thinning, development of pyogenic granuloma or inclusion cyst), further progression of the lesion in lymphoma and pemphigoid, and/or recurrence of two pterygia (4/10), incomplete excision in a case of nonspecific inflammatory lesion (1/10) and enucleation in one case because of high-grade carcinoma with sebaceous differentiation. It is difficult to compare the overall outcome with other studies because they mostly discuss outcomes in relation to specific entities. However, in their large study of 5002 benign and malignant conjunctival lesions, Shields et al. reported one enucleation and five exenterations in cases with sebaceous gland carcinoma [4].

The limitation of this study is the relatively small number of malignant conjunctival lesions studied histopathologically, possibly because of other available offered treatment modalities for such patients.

## Conclusions

In conclusion, this study showed that the most commonly biopsied lesions in a Saudi cohort were mostly benign, falling within the category of fibrodegenerative lesions (pterygia or pinguecula) followed by benign nevi. Malignant conjunctival lesions presented within a statistically significant shorter duration, and they were all either epithelial or hematolymphoid, with no cases of melanoma. The bulbar conjunctiva nasally and temporally were the most frequent sites of involvement. Males were affected more frequently by conjunctival lesions than females, and this predilection was statistically significant in terms of the frequency of fibrodegenerative lesions in adults due to the local lifestyle. This is a universal finding in concordance with other studies elsewhere, except for Iran. On the other hand, OSSN was found to be equally prevalent among males and females compared to being observed with double frequency among males three decades ago, probably because of the shift in females’ culture and lifestyle. Melanocytic lesions are more prevalent in younger age groups. The preoperative diagnosis was not necessarily accurate in all cases; however, it is more likely to accurately diagnose benign conjunctival lesions than malignant ones. Similarly, benign conjunctival lesions are generally expected to have a more favorable outcome.

This study proves the wide variation in the reported prevalence and clinicopathological characteristics of conjunctival lesions in different areas of the world and depends on the variability of study designs and inclusion criteria. In our Saudi population, the findings reflected our geographical and environmental characteristics in addition to the cultural lifestyle. This information will be useful for future preventive and therapeutic studies on these important lesions.

## Data Availability

The data that support the findings of this study are available from the corresponding author upon reasonable request.

## Abbreviations

OSSN: Ocular surface squamous neoplasia.
SCC: Squamous cell carcinoma.
PAM: Primary acquired melanosis.
IHC: Immunohistochemical.
WHO: World Health Organization.
BRLH: Benign reactive lymphoid hyperplasia.
CIN: Conjunctival intraepithelial neoplasm.
ENMZL: Extranodal marginal zone lymphoma
MALT: Mucosal-associated lymphoid tissue
